# TWIST1 induces phenotypic switching of vascular smooth muscle cells by downregulating p68 and microRNA‐143/145

**DOI:** 10.1002/2211-5463.13092

**Published:** 2021-02-03

**Authors:** Jing Zhang, Jie‐Ru Guo, Xian‐Li Wu, Xia Wang, Zhi‐Ming Zhu, Yong Wang, Xia Gu, Ye Fan

**Affiliations:** ^1^ Department of Respiratory Disease Xinqiao Hospital Third Military Medical University Chongqing China; ^2^ Department of Hypertension and Endocrinology Daping Hospital Third Military Medical University Chongqing China; ^3^ Department of Cardiovascular Surgery Xinqiao Hospital Third Military Medical University Chongqing China; ^4^ Department of Pathology First Affiliated Hospital of Guangzhou Medical University Guangzhou China

**Keywords:** microRNA‐143/145, p68, phenotypic switching, TWIST1, vascular smooth muscle cell

## Abstract

TWIST1 is an important basic helix‐loop‐helix protein linked to multiple physiological and pathological processes. Although TWIST1 is believed to be involved in vascular pathogenesis, its effects on homeostasis of smooth muscle cells (SMCs) remain poorly understood. Here, we show that TWIST1 protein levels were significantly elevated during SMC phenotypic switching *in vivo* and *in vitro*. TWIST1 overexpression promoted phenotypic switching of SMCs, while siRNA targeting of TWIST1 prevented cell transition. Mechanistically, TWIST1 decreased the level of microRNA‐143/145, which governs smooth muscle marker gene transcription. In addition, TWIST1 repressed p68 mRNA and protein expression, a crucial modulator of SMC behavior and microRNA biogenesis. Our co‐immunoprecipitation assay demonstrated a previously unrecognized molecular interaction between TWIST1 and p68 protein. Finally, we found that TWIST1 triggered SMC phenotypic switching and suppressed microRNA‐143/145 expression by promoting the proteasomal degradation of p68. These data suggest a novel role of TWIST1 in the regulation of SMC homeostasis by modulating p68/microRNA‐143/145 axis.

## Graphical abstract

Increased TWIST1 expression leads to the suppression of the DDX5/microRNA‐143/145 pathway in VSMC, which induces phenotypic switching of VSMC and promotes the progression of vascular remodeling. Therefore, TWIST1 might be a attractive therapeutic target for inhibiting phenotype‐related vascular disorders.

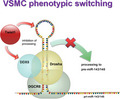

**Abbreviations**
BSA: bovine serum albumin
DAPI: 4',6‐diamidino‐2‐phenylindole
FBS: fetal bovine serum
GFP: green fluorescence protein
PCR: real‐time polymerase chain reaction
PDGF: platelet‐derived growth factor
PVDF: polyvinylidene difluoride membrane
siRNA: specific small interfering RNA
SMA: alpha‐smooth muscle actin
SMC: smooth muscle cell
SMMHC: smooth muscle myosin heavy chain

Smooth muscle cells (SMCs) are the major component of vascular wall, which play a crucial role in a great variety of arterial diseases. Different from terminally differentiated cells, SMCs possess apparent plasticity that allows their transition in response to environmental stimuli, which is characterized by alternations in protein expression, morphology, and biological behavior [1]. In normal condition, mature SMCs have a differentiated/contractile phenotype, characterized by the expression of a unique repertoire of contractile proteins, such as smooth muscle myosin heavy chain (SMMHC), alpha‐smooth muscle actin (SMA), calponin, and tagln. These cells are elongated and display spindle‐shape morphology. However, SMCs could undergo phenotypic switching to a dedifferentiated/synthetic state when subjected to multiple stimulation. The process is characterized by decreased expression of smooth muscle markers, enhanced SMC proliferation, altered cellular function, and changed shape from spindle‐like to polygonal. Pathological SMC transition is a fundamental process for the development of various cardiovascular disorders, such as atherosclerosis, aneurysm, and hypertension [2, 3, 4]. Despite its essentiality in vascular regulation, the molecular mechanisms underlying SMC phenotypic switching remain incompletely understood.

TWIST1 is a basic helix‐loop‐helix transcription factor implicated in cell lineage determination and differentiation. Mutation or abnormal expression of TWIST1 gene is closely associated with several diseases, such as breast cancer, Sézary syndrome, and Chotzen syndrome [5, 6]. Notably, recent reports have suggested a potential role of TWIST1 in the pathogenesis of vascular disorders [7, 8, 9]. Genome‐wide association study has identified TWIST1 as a causal gene for common vascular diseases [7]. Mahmoud and colleagues reported that blood flow‐induced shear stress increases TWIST expression and subsequently promotes vascular abnormality by inducing endothelial cell proliferation and inflammation [8]. In our recent study, SMC‐specific depletion of TWIST1 protected against pulmonary vascular remodeling, thus implicating a detrimental role of TWIST1 in both systemic and pulmonary circuits [9].

Despite human studies and functional animal experiments have suggested the potential participation of TWIST1 in vascular disorders, little information is presently available regarding its role in SMC homeostasis. As one of the pernicious transcriptional regulators involved in cellular differentiation, we hypothesized that TWIST1 might contribute to SMC transition therefore exerts promotory effects on cell misbehavior and vascular disease development. In this study, we identified TWIST1, whose expression is greatly increased in dedifferentiated/synthetic SMCs, as a novel modulator of SMC phenotypic switching via downregulation of p68 and microRNA‐143/145.

## Materials and methods

Details of materials and experimental procedures are available in the Supporting Information.

### Animal experiment

All animal experiments were approved by Animal Care and Use Committee of Third Military Medical University. Male Sprague‐Dawley rats weighing 220 to 250 g were used in these experiments, as previously described [10]. They were anesthetized by the intraperitoneal injection of 1% pentobarbital sodium (Sigma‐Aldrich, St. Louis, MO, USA, 5 mg/100 g body weight). After the rats were anesthetized, the left common carotid arteries were carefully exposed through a midline cervical incision. Blood flow transiently stooped by occluding the arteries with vascular clamps, and then, a small arteriotomy was made around the external carotid artery. Afterward, the vessels were completely injured near the carotid bifurcation using a balloon embolectomy catheter (1.5F, Cordis, Miami Lakes, FL) through the incision hole. Once injured, the wounded skin was closed with a single suture. The rats were carefully looked after in the Animal Center of Third Military Medical University. The rats were sacrificed at indicated time points with intraperitoneal injection of 1% pentobarbital sodium (30mg/100 g body weight). Anesthesia was monitored by assessment of pain and corneal reflexes. Isolation of the carotid arteries was started after both reflexes completely disappeared. The carotid arteries were obtained and cleaned for the predesigned assays. Carotid arteries from sham‐operated rats served as control.

### Cell culture

Human aortic SMCs were purchased from the American Type Culture Collection (Manassas, VA, USA). Cells were cultured in F12 Kaighn’s medium (HyClone, Logan, UT, USA) supplemented with 10% fetal bovine serum (FBS, Gibco, Grand Island, NY, USA). SMCs were used at passages 3 through 7.

### Real‐time polymerase chain reaction (PCR)

Total RNA was extracted from tissues and confluent SMCs with TRIzol reagent (TaKaRa, Tokyo, Japan) following the manufacturer’s protocol. The RNA (1000 nmol) was then subjected to reverse transcription using the PrimeScript RT reagent Kit with gDNA Eraser (TaKaRa). Afterward, SYBR Green (TaKaRa)‐based real‐time PCR was carried out by Applied Biosystems 7500 real‐time PCR system (Applied Biosystems, Foster City, CA). Real‐time PCR was performed in 20uL reactions with 10 pmol primers produced by Shangon Biotech (Shanghai, China), and the relative quantitative analysis of the change in expression levels was obtained using the comparative Ct method and normalized to the corresponding control. The specific sequences of the primers used were listed in Table 1.

**Table 1:** Specific sequences of the primers

Primer name	Primer sequences	Annealing temperature/fragment size	Corresponding reference RNA
miR‐143	For: ssD809230846 Rev: ssD089261711 (RiboBio Co., Ltd)	60°C/78bp	U6
miR‐145	For: ssD809231411 Rev: ssD089261711 (RiboBio Co., Ltd)	60°C/81bp	U6
U6	For: ssD0904071006 Rev: ssD0904071007 (RiboBio Co., Ltd)	60°C/76bp	‐
p68	For: 5’‐GCACAGCACAAGAGGTGGAA‐3’ Rev: 5’‐TCCCTGAGCTTGAATAGCAGT‐3’	60°C/170bp	β‐actin
β‐actin	For: 5’‐GACTTAGTTGCGTTACACCCTTTCT‐3’ Rev: 5’‐TGTCACCTTCACCGTTCCAGT‐3’	60°C/157bp	‐

### Western blot analysis

SMCs were washed twice with precold PBS and harvested on ice in RIPA (Beyotime, Wuhan, China) supplemented with 1% phenylmethlsulfonyl fluoride (Beyotime). All tissue samples were frozen with liquid nitrogen and homogenized, and then lysed as previously described. The whole protein samples were diluted with 5 × SDS/PAGE loading buffer and boiled 5 min. Equal amounts of protein were loaded on SDS/PAGE gels. Constant voltage was used in this step (120V running, 80V stacking). Then, proteins were transferred onto a polyvinylidene difluoride membrane (PVDF, Millipore, Etten‐Leur, Netherlands) by electroblotting at constant electronic of 150 mA, followed by blocking with 5% bovine serum albumin (BSA) (Boster, Wuhan, China) for 1 h at room temperature. The membranes were incubated in their primary antibodies overnight at 4°C (Table 2, Dilutions: antibodies against TWIST1 [1 : 300], SMMHC [1 : 500], SMA [1 : 1000], calponin [1 : 500], tagln [1 : 1000], GAPDH [1 : 500], p68 [1 : 1000], and β‐actin [1 : 1000]). The following day, the blots were washed five times (3 min per time) with Tris‐Buffered Saline with Tween‐20 (Beyotime); signals were detected by appropriate secondary horseradish peroxidase‐conjugated antibodies (1 : 5000) (Abcam, Cambridge, MA, USA) for 1 h at room temperature, and visualized via enhanced chemiluminescence (Amersham International, Buckinghamshire, UK). Optical density of bands was quantified by Quantity One software and normalized with loading control.

**Table 2:** Antibodies for immunoblot analyses

Antibody	Manufacturer	Catalog	Source of species	MW (kDa)
TWIST1	CST	46702	rabbit	26
β‐actin	Boster	BM0627	mouse	42
SMMHC	PROTEINTECH	21404‐1‐AP	rabbit	200
SMA	ABCAM	Ab7817	mouse	42
calponin	PROTEINTECH	13939‐1‐AP	rabbit	33
tagln	PROTEINTECH	10493‐1‐AP	rabbit	22
GAPDH	CST	5174S	rabbit	37
p68	CST	9877	rabbit	68

### Lentivirus production and transfection

Lentivirus‐TWIST1 siRNA and control lentivirus carrying green fluorescence protein (GFP) were produced by Invitrogen (Carlsbad, CA, USA). TWIST1 overexpressed lentivirus and the corresponding GFP control were obtained from GeneChem (Shanghai, China). p68 siRNA and the corresponding GFP control were obtained from Hanbio Biotechnology. SMCs were seeded in 24‐well plate at a density of 3 × 10^4^ and cultured in F12K containing 10% FBS 24 h prior to infection. After rinsing with PBS, the lentivirus and the corresponding controls, mixed in F12K, were added into SMCs, supplemented with 0.5% polybrene. Twenty‐four hours later, the medium was replaced with fresh complete growth media, and the transfection efficiency was measured by detecting GFP expression under fluorescence microscope. The transfection efficiency of more than 95% was regarded as successful. Real‐time RT–PCR and western blot were further used to confirm the results of the transfection.

### Immunofluorescence

SMCs were seeded onto coverslips in 6‐well plates for 24hrs prior to serum starvation (approximately 5 × 10^5^ cells/well). SMCs on the coverslips were fixed with 4% ice‐cold paraformaldehyde for 15 min at room temperature, permeabilized with 0.5% Triton X‐100, and rinsed with PBS. After washing with PBS for three times, the cells were blocked with 5% BSA for 20 min. The sections were first incubated with anti‐TWIST1 antibody overnight at 4 °C. Then, secondary FITC‐conjugated antibody was added. For detection of cytoskeleton remodeling, rhodamine phalloidin was used to stain F‐actin. In all immunofluorescence‐based assays, the nuclei were counterstained with 4',6‐diamidino‐2‐phenylindole (DAPI). The stained cells were visualized with an Olympus confocal microscope. For immunofluorescence analysis of vessels, after deparaffinization, tissue samples were permeabilized with 1% Triton X‐100 and then blocked with 5% BSA for 20 min, at last, with primary antibodies against TWIST1 and SMA in PBS overnight at 4 °C. Secondary antibodies were incubated for 1 h at room temperature.

### Co‐immunoprecipitation

The whole protein extracts of SMCs were prepared using the special lysis buffer (Cell Signaling Technology no. #9803, Danvers, MA, USA) for immunoprecipitation. The lysates were centrifuged, and protein concentrations were measured (BCA kit) and immunoprecipated at 4°C by gently rocking overnight with the protein A/G magnetic beads (Cell Signaling Technology) prebound to anti‐TWIST1 or anti‐p68 antibodies prior to bind with the immunoprecipitates. Precipitated proteins were washed 5 times with PBS, boiled in 2 × sample loading buffer, and separated by 12% SDS/PAGE. The separated protein was then transferred to PVDF membrane and immunoblotted with anti‐TWIST1 or anti‐p68 antibody, respectively. Rabbit normal IgG (Cell Signaling Technology) served as negative control.

### Statistical analysis

All data are expressed as mean ± SEM from three or more independent experiments and analyzed with PASW Statistic 21 (SPSS Inc., Chicago, IL, USA). Data between two groups were compared using two‐tailed Student’s *t*‐test. Multiple comparisons utilized one‐way ANOVA followed by Student–Newman–Keuls test. *P* < 0.05 was considered as significant difference.

## Results

### Association of TWIST1 and SMC phenotypic switching

**Fig. 1:**
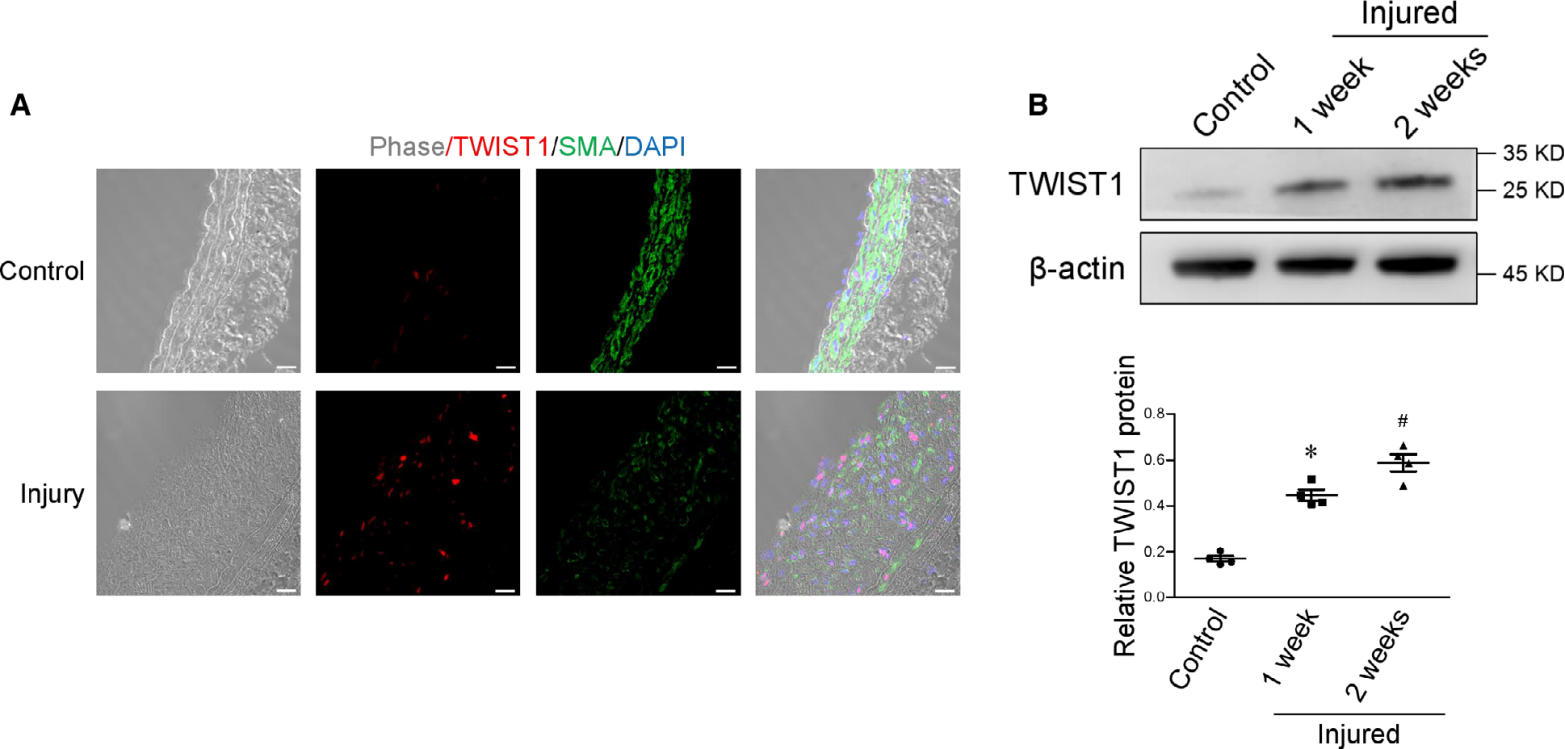
TWIST1 expression is increased in the blood vessels in neointimal injury model. (A) Phase and immunofluorescence confocal images revealed TWIST1 (red) expression in control and injured rat carotid arteries. Anti‐SMA staining for SMA (green). Cell nuclei were stained with DAPI (blue). Scale bar = 20 μm. (B) western blots of TWIST1 from sham‐operated vessels and balloon‐injured carotid arteries at 7 and 14 days after operation, *P < 0.05 vs control, ^#^ *P* < 0.05 vs injured 1 week. Statistical analysis was performed using one‐way ANOVA followed by Student–Newman–Keuls test. Data were reported as the mean ± SEM

To determine whether TWIST1 is involved in smooth muscle phenotypic modulation, we evaluated its expression in the vessels from the rat carotid injury model. As shown in Figure 1A, TWIST1 staining was increased in the media region of the pathological vessels, which was associated with suppressed SMC differentiation marker—SMA, as compared with control vessels. Accordingly, western blot confirmed a significant upregulation of TWIST1 protein in the injured arteries, suggesting a negative correlation between TWIST1‐ and SMC‐specific markers in vivo (Figure 1B).

**Fig. 2:**
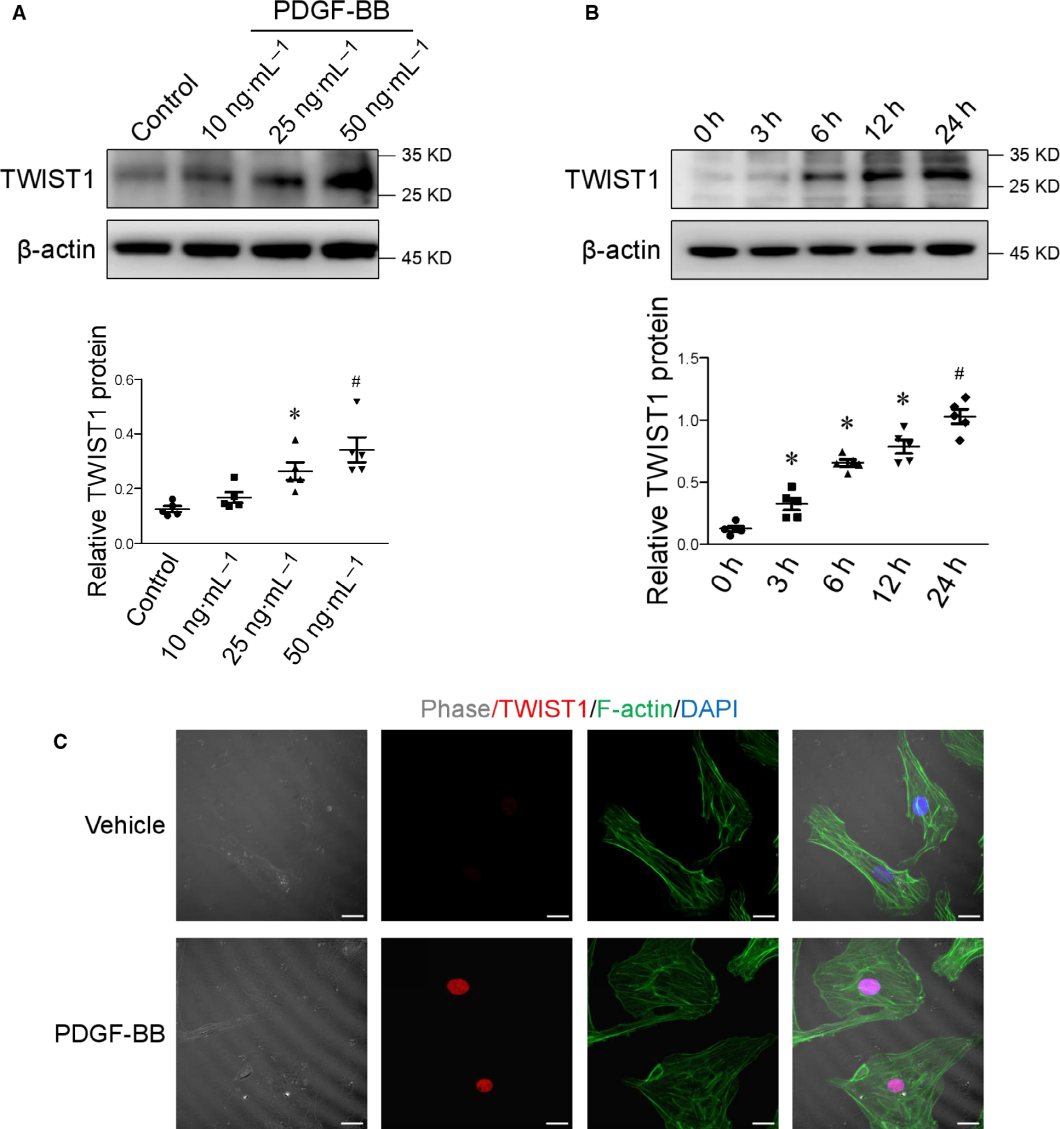
TWIST1 level is elevated in dedifferentiated/synthetic SMCs. (A) Representative western blot of TWIST1 protein in SMCs challenged with different concentrations of PDGF‐BB. *P < 0.05 vs control, ^#^ *P* < 0.05 vs 25ng·mL^−1^. (B) Cultured SMCs were treated with 25ng·mL^−1^ PDGF‐BB. Cell lysates were obtained at indicated time points and probed with antibodies against TWIST1. *P < 0.05 vs 0 h, ^#^ *P* < 0.05 vs 12 h. (C) Phase and immunofluorescence confocal images revealed the effects of 25ng·mL^−1^ PDGF‐BB incubation on TWIST1 (red) expression in SMCs. Rhodamine phalloidin for F‐actin (green). Cell nuclei were stained with DAPI (blue). Scale bar = 20 μm. Statistical analyses were performed using one‐way ANOVA followed by Student–Newman–Keuls test. Data were reported as the mean ± SEM.

To further verify the above observations in vitro, cultured human aortic SMCs were stimulated with platelet‐derived growth factor (PDGF)‐BB, which is a widely used approach for SMC differentiation study (Figure S1). The reduced expression of smooth muscle markers (SMMHC, SMA, calponin, and tagln) confirmed SMC phenotypic switching due to PDGF‐BB treatment. As shown in Fig. 2A,B, PDGF‐BB both dose‐ and time‐dependently enhanced TWIST1 expression in human vascular SMCs. Similarly, we observed an increase in TWIST1 level in PDGF‐BB‐induced dedifferentiated/synthetic SMCs, as evidenced by the morphological and cytoskeletal alternation (Fig. 2C). Therefore, the data suggest that TWIST1 is closely associated with the expression of smooth muscle markers during SMC phenotypic switching.

### TWIST1 induces phenotypic switching of SMC

**Fig. 3:**
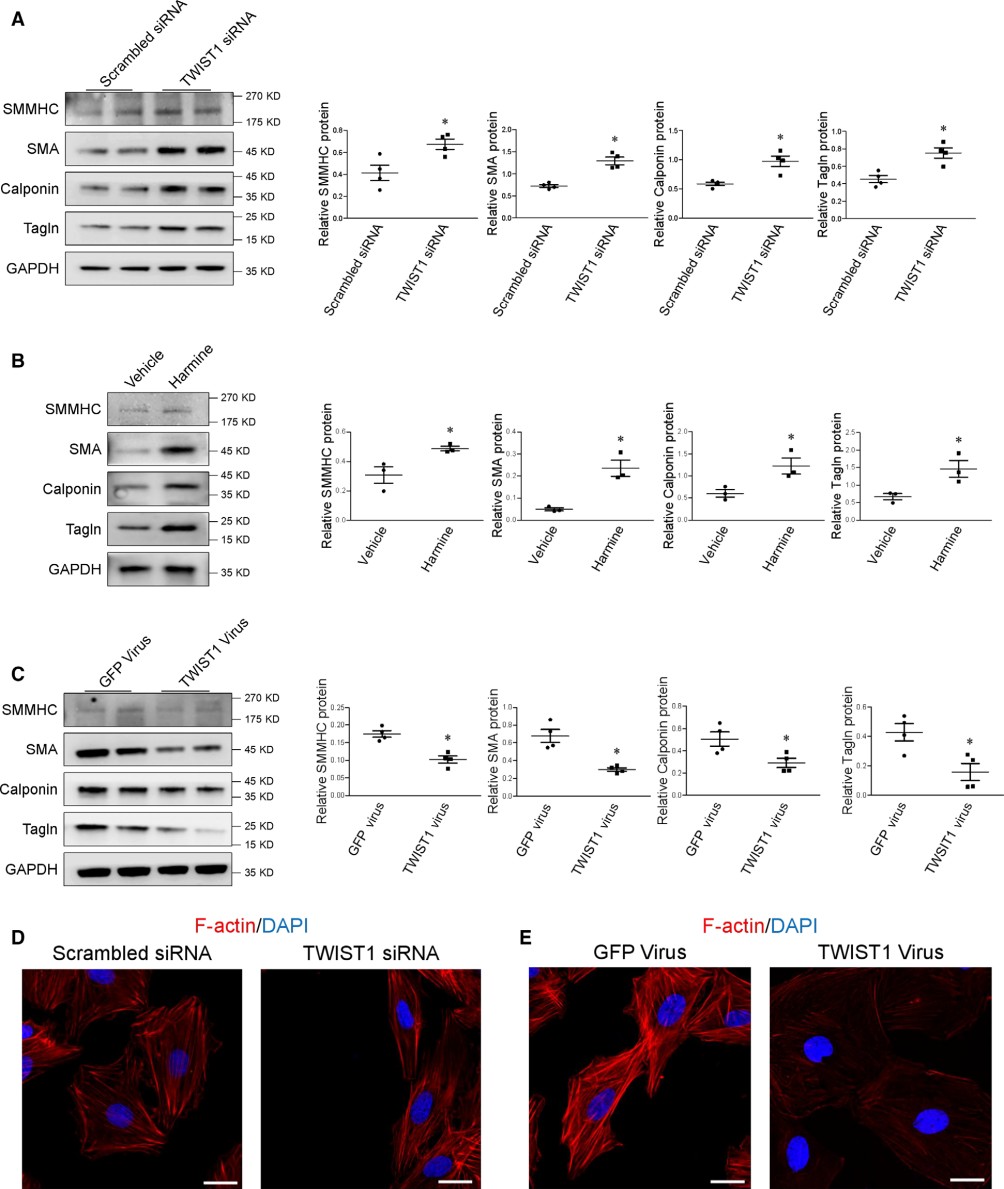
TWIST1 induces SMC phenotypic switching. (A) Protein levels of SMMHC, SMA, calponin, and tagln in SMCs transfected with scrambled or TWIST1‐specific siRNA, *P < 0.05. (B) SMCs were treated with either vehicle or harmine for 24hrs. Expression of SMMHC, SMA, calponin, and tagln protein was measured by western blot. *P < 0.05. (C) Representative immunoblots of SMMHC, SMA, calponin, and tagln protein expression in control or TWIST1 overexpressing SMCs. *P < 0.05. (D) and (E) Immunofluorescence confocal images revealed F‐actin (red) in SMC transfected with TWIST1‐specific siRNA (D) and TWIST1 virus (E). Cell nuclei were stained with DAPI (blue). Scale bar = 20 μm. Statistical analyses were performed using two‐tailed Student’s *t*‐test. Data were reported as the mean ± SEM

In an attempt to identify the potential role of TWIST1 in SMC phenotypic switching, specific small interfering RNA (siRNA) was used to knockdown TWIST1 in human aortic SMCs. Control cells were transfected with lentivirus containing scrambled control. We found that suppression of TWIST1 increased SMC‐specific markers, as evaluated by western blot (Fig. 3A). Similarly, the application of harmine, a small molecular inhibitor targeting TWIST1, significantly enhanced SMC marker protein expressions (Fig. 3B).

Next, we examined the effects of TWIST1 overexpression on SMC homeostasis. For these experiments, lentivirus containing GFP or TWIST1‐GFP constructs was applied. We showed that enforced TWIST1 expression strongly suppressed the expressions of SMC contractile proteins (Fig. 3C). Accordingly, TWIST1 siRNA promoted a spindle‐like cell shape, while overexpression induced significant morphological change in human vascular SMCs (Fig. 3D,E). Thus, these results further support TWIST1 as being an important regulator of SMC phenotypic transition.

### MicroRNA‐143/145 expression in SMCs is repressed by TWIST1

**Fig. 4:**
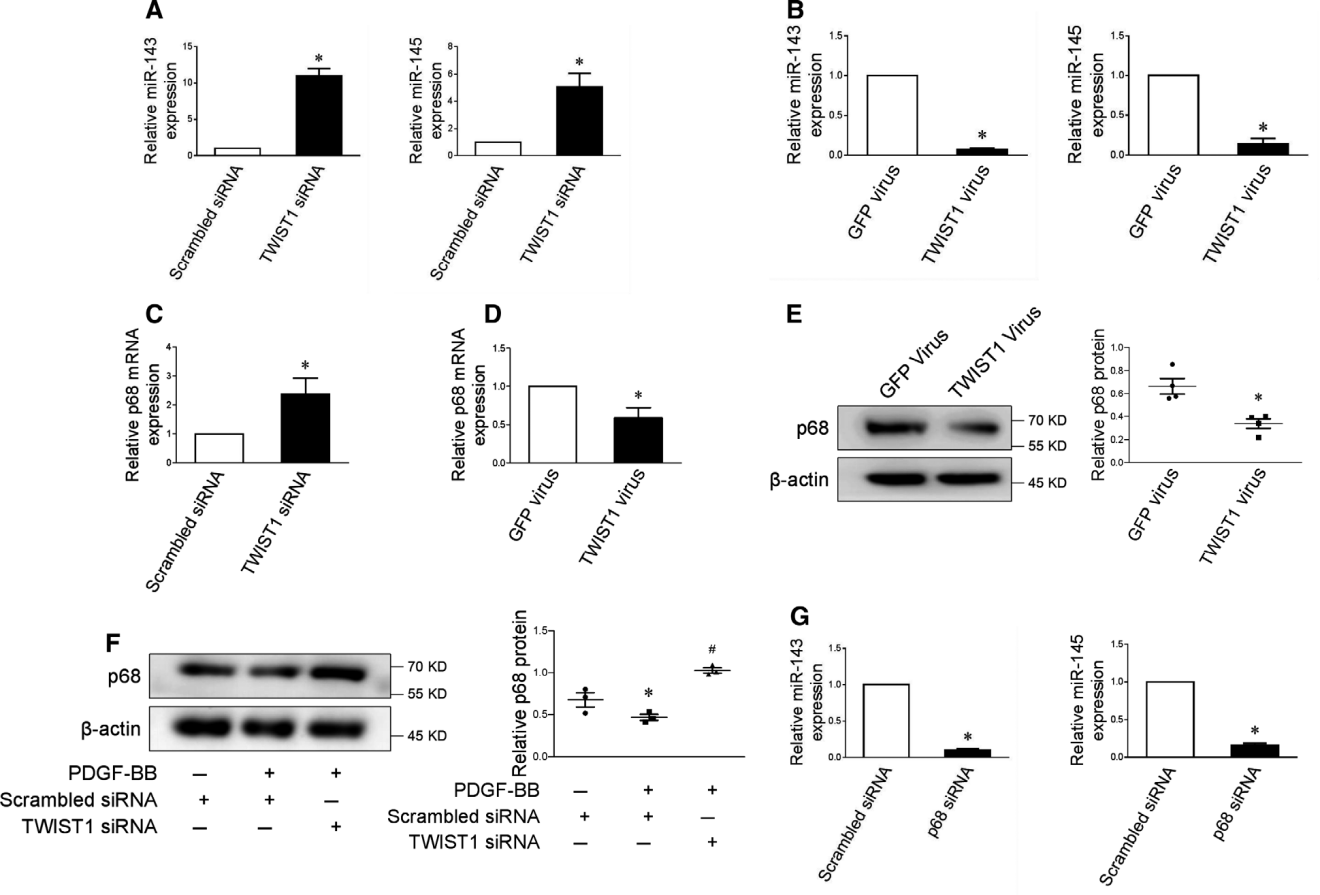
TWIST1 reduces microRNA‐143/145 and p68 expression SMCs. (A) Quantitative real‐time PCR analysis of microRNA‐143/145 in SMCs transfected with scrambled or TWIST1‐specific siRNA, *n* = 3, statistical analyses were performed using two‐tailed Student’s *t*‐test. *P < 0.05. (B) Quantitative real‐time PCR analysis of microRNA‐143/145 in control or TWIST1 overexpressing SMCs. *n* = 3, statistical analyses were performed using two‐tailed Student’s *t*‐test. *P < 0.05. (C) Quantitative real‐time PCR analysis of p68 mRNA in SMCs transfected with scrambled or TWIST1‐specific siRNA, *n* = 5, statistical analysis was performed using two‐tailed Student’s *t*‐test. *P < 0.05. (D) Quantitative real‐time PCR analysis of p68 mRNA in control or TWIST1 overexpressing SMCs. *n* = 4, statistical analysis was performed using two‐tailed Student’s t‐test. *P < 0.05. (E) p68 protein levels in SMCs transfected with control or TWIST1 virus, statistical analysis was performed using two‐tailed Student’s *t*‐test. *P < 0.05. (F) Representative blots of p68 protein expression in SMCs transfected with scrambled or TWIST1‐specific siRNA receiving additional PDGF‐BB, statistical analysis was performed using one‐way ANOVA followed by Student–Newman–Keuls test. *P < 0.05 vs scrambled siRNA, ^#^ *P* < 0.05 vs scrambled siRNA + PDGF‐BB. (G) Quantitative real‐time PCR analysis of microRNA‐143/145 in SMCs transfected with scrambled or p68‐specific siRNA, *n* = 4, statistical analyses were performed using two‐tailed Student’s *t*‐test. *P < 0.05. All data were reported as the mean ± SEM.

After observing the suppressive effects of TWIST1 on smooth muscle proteins, we attempted to explore the potential mechanisms involved. Although prior study suggested the potential binding sites for bHLH proteins within SMA promoter regions, our bioinformatic search indicated no direct interaction between TWIST1 and the promoters of other SMC differentiation marker genes (http://cistrome.org/db/#/) ^11^. MicroRNAs, modulating various biological processes by inducing target mRNA degradation or blocking translation, have been recently proved as potent regulators of SMC differentiation and biological behavior [11, 12, 13]. MicroRNA‐143/145 is one of the first identified micoRNA clusters participating in the phenotypic modulation of SMC; thus, we wondered whether it is involved in the process of TWIST1‐induced SMC phenotypic switching [14, 15]. Prior studies have shown significantly reduced microRNA‐143/145 expression in SMCs from injured arteries, while overexpression of microRNA‐143/145 is sufficient to promote differentiation and inhibit proliferation of SMCs [15, 16]. Of note, we found that TWIST1 downregulation led to prominent increase in microRNA‐143/145 in SMCs, as compared with control (Fig. 4A). In contrast, enforced TWIST1 expression significantly repressed microRNA‐143/145 level (Fig. 4B).

Our previous study showed that p68 is pivotal for maintaining the contractile phenotype of SMCs, which is also one of the major proteins responsible for the canonical microRNA biogenesis [10]. Similar to the prior microRNA‐143/145 report, the expression of p68 was decreased in smooth muscle of the pathological vessels (Fig. S2). To determine whether p68 is involved in the negative regulation of SMC phenotype and microRNA‐143/145 by TWIST1, we first examined the potential impacts of TWIST1 on p68 expression (Fig. 4C,D). The data showed that knockdown of TWIST1 efficiently improved p68 mRNA level in SMCs, whereas lentivirus‐mediated enforced expression of TWIST1 markedly repressed p68. Although TWIST1 is known as a transcription factor, the chip‐sequencing datasets revealed no close interaction between TWIST1 and the promoter of p68, indicating a potentially indirect inhibition of p68 mRNA level (http://cistrome.org/db/#/). In agreement with the findings from the mRNA study, western blots confirmed a negative correlation between TWIST1 and p68 (Fig. 4E,F). Furthermore, we found that p68 knockdown markedly reduced microRNA‐143/145 expression (Fig. 4G). Based on the results, we speculated upon the possibility of p68 protein as a downstream mediator of TWIST1‐induced suppression of microRNA‐143/145 and SMC transition.

### TWIST1 suppresses microRNA‐143/145 in a p68‐dependent manner

**Fig. 5:**
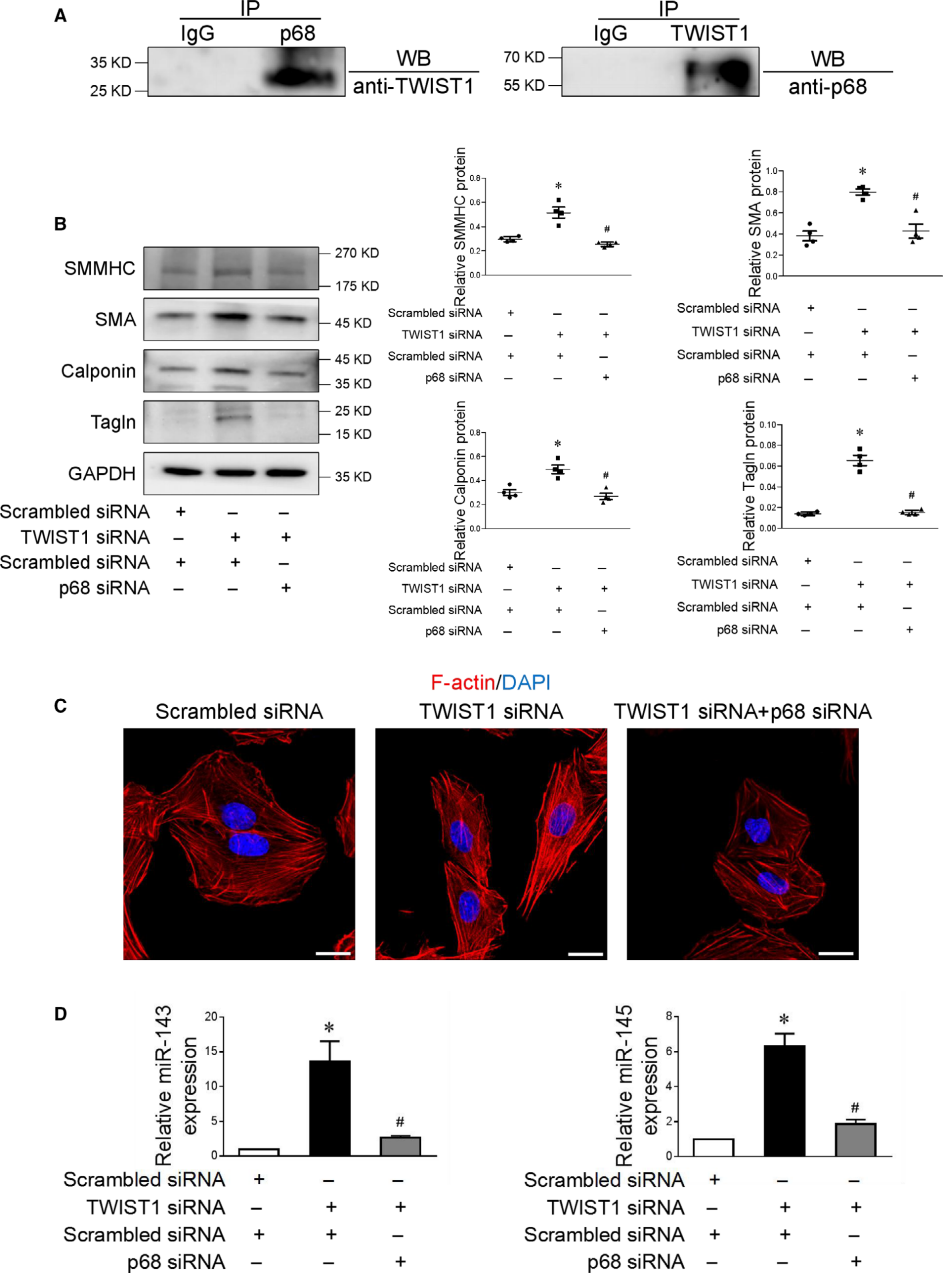
TWIST1 decreases microRNA‐143/145 expression by downregulation of p68. (A) Co‐immunoprecipitation assay of TWIST1 and p68 in SMCs. *n* = 3. (B) Representative immunoblots of SMMHC, SMA, calponin, and tagln protein in SMCs transfected with TWIST1‐specific siRNA receiving either scrambled or p68‐specific siRNA, *P < 0.05 vs scrambled siRNA, ^#^ *P* < 0.05 vs TWIST1 siRNA. (C) Immunofluorescence confocal images revealed F‐actin (red) in SMCs transfected with TWIST1‐specific siRNA receiving either scrambled or p68‐specific siRNA. Cell nuclei were stained with DAPI (blue). Scale bar = 20 μm. (D) Quantitative real‐time PCR analysis of microRNA‐143/145 in SMCs transfected with TWIST1‐specific siRNA receiving either scrambled or p68‐specific siRNA. *n* = 4, *P < 0.05 vs scrambled siRNA, ^#^ *P* < 0.05 vs TWIST1 siRNA. Statistical analysis was performed using one‐way ANOVA followed by Student–Newman–Keuls test. Data were reported as the mean ± SEM.

To further verify the potential link between TWIST1 and p68, we performed co‐immunoprecipitation assay using the detergent extracts prepared from human aortic SMCs. As shown in Fig. 5A, western blot analysis of immunoprecipitates demonstrated a specific p68 band brought down by TWIST1 antibody, but not by control IgG. In accordance with this observation, co‐immunoprecipitation with anti‐p68 antibody revealed that p68 also precipitated TWIST1. Thus, TWIST1 was capable of binding to p68. Prior study showed that TWIST1 could regulate the activity of proteasome system [17]. Thus, the proteasome inhibitor MG132 was applied to determine whether the inhibitory effect of TWIST1 on p68 was proteasome‐mediated. We demonstrated that suppression of p68 protein by TWIST1 overexpression in SMCs was partiality blocked at the presence of MG132, indicating that TWIST1 might directly target p68 and promote its proteasomal degradation (Fig. S3).

To further assess the contribution of p68 to the negative role of TWIST1 in SMC differentiation, we transfected SMCs with TWIST1 siRNA lentivirus plus either scrambled or p68 siRNA lentivirus. Importantly, we found that knockdown of p68 abolished the protective effects of TWIST1 depletion on SMC homeostasis, as evidenced by western blot and immunofluorescence staining (Fig. 5B,C). In accordance with the SMC phenotype study, transfection of p68 siRNA lentivirus but not scrambled control largely inhibited TWIST1 silencing‐induced elevation of microRNA‐143/145 level, suggesting that TWIST1‐guided regulation of SMC transition and microRNA‐43/145 expression might be mediated by p68 (Fig. 5D). Taken together, our data present evidence that TWIST1‐induced SMC phenotypic switching is attributable to inhibition of p68‐dependent microRNA‐143/145 expression.

## Discussion

Although prior studies have indicated a role of TWIST1 in vascular disease and endothelial function, its contribution to SMC transition remains unclear. To our knowledge, this is the first study demonstrating that TWIST1, a key regulator of endothelial/epithelial to mesenchymal transition, promoted SMC phenotypic switching that is necessarily required for the development of multiple vascular abnormalities, and intimately linked with SMC behavior and function. The underlying mechanism of this process is that TWIST1 directly binds to p68 protein and decreases its expression. TWIST1 downregulates microRNA‐143/145 expression through repressing p68, which is the molecular key to switch the phenotype of SMCs. Overall, the data showed a previously unknown role of TWIST1 in SMC homeostasis regulation.

TWIST1, a critical embryonic transcription factor, was initially discovered in Drosophila and extremely important for the early development of mesoderm formation [18, 19]. It belongs to the basic helix‐loop‐helix transcriptor family, which plays significant roles in various physiological and pathophysiological processes. In humans, TWIST1 mutation leads to Saethre‐Chotzen syndrome, a genetic disorder characterized by the premature fusion of certain skull bones. TWIST1 is well‐known for its essential role in tumor development, metastasis, and chemoresistance in a variety of cancers [20, 21]. Intriguingly, recent evidence suggests TWIST1 acting as a crucial mediator of arterial disease development and vascular cell misbehavior [7, 8, 9].

In spite of the efforts evaluating the contributions of TWIST1 to vascular angiogenesis and pathogenesis, most of them payed their attentions to its proproliferative property, and little information is currently available regarding its effects on SMC homeostasis that is the basis for the development and progression of various arterial disorders. Besides, despite the close relationships between TWIST1 and a wide range of arterial abnormalities, a universal interpretation of its extensive and detrimental effects in the world of vascular disease is absence. Therefore, we first determined the change in TWIST1 expression during SMC phenotypic transition, which showed a markedly elevated TWIST1 level in both injured rat carotid artery and PDGF‐BB‐treated dedifferentiated/synthetic SMCs. Importantly, inhibition of TWIST1 through either specific siRNA or pharmacological inhibitor prevented SMC phenotypic switching. Likewise, overexpression of TWIST1 resulted in the transition of SMCs to a synthetic state, thus reinforced the role of TWIST1 in SMC phenotypic switching, and made us to further explore the mechanism involved.

Loss of smooth muscle markers is the hallmark of SMC phenotypic switching. As an important regulator of targeted gene transcription, TWIST1 has been shown to inhibit SMA expression by binding to and inactivating SMA promoter [22]. However, our ChIP‐sequencing database search revealed no direct interplay between TWIST1 and the promoters of other SMC differentiation markers; thus, its transcription regulatory property might not fully explain the universal suppressive effects of TWIST1 on smooth muscle genes. MicroRNAs have recently received much attention for their ability in tempering gene expression [23, 24, 25]. In particular, they can act as crucial modulators of SMC phenotypic transition and proliferation [26, 27]. Several microRNAs, including microRNA‐143/145, microRNA‐663, and microRNA‐133, have a demonstrated role in SMC phenotypic change [28, 29, 30]. Notably, we showed that TWIST1 inhibition increased microRNA‐143/145 expression, while overexpression of TWIST1 downregulated its level, indicating that the function of TWIST1 on SMC plasticity might be mediated by microRNA‐143/145.

In delineating how TWIST1 triggered SMC transition and repressed microRNA‐143/145, we focused our attention on p68, a prototypic member of the DEAD box family that is a vital regulator of both SMC differentiation and microRNA processing [31]. The present study showed that TWIST1 overexpression inhibited p68 protein and mRNA levels, while TWIST1 inhibition recruited PDGF‐BB‐induced downregulation of p68 expression. In addition, knockdown of p68 significantly decreased microRNA‐143/145, which is in accordance with previous reports demonstrating its potent capacity in regulating microRNA [32]. Although TWIST1 is known for its transcription regulatory capacity, no evidences support the interaction between TWIST1 and p68 promoter, thus implicating a potentially indirect regulation of p68 mRNA level. The co‐immunoprecipitation experiment revealed a de novo interplay between TWIST1 and p68. In addition, the data suggested that TWIST1 induces the proteasomal degradation of p68. Thus, TWIST1 might be a molecular partner of p68 and directly impact its expression and biological function, which leads to SMC transition. Importantly, transfection of p68 siRNA inhibited the protective effects of TWIST1 inhibition on SMC phenotypic switching and microRNA‐143/145 expression, implying that TWIST1‐induced SMC phenotypic modulation is, at least partially, through downregulation of microRNA‐143/145 via repressing p68.

### Limitation

Our study has several limitations. First, the in vitro study could be biased by the switch of SMCs from the contractile to synthetic phenotype when they were passaged. However, we have tried our best to use SMC from the same passage in each experiment, which might reduce this bias. Second, there were no relevant animal experiments assessing the impacts of TWIST1 on SMC phenotypic switching. The in vivo regulatory effects of TWIST1 on SMC homeostasis still need further research.

## Conclusions

In conclusion, our work defines a previously unreported role of TWIST1 in modulating SMC differentiation, through a novel mechanism that it inhibits microRNA‐143/145 in a p68‐dependent manner. TWIST1 might be a potential therapeutic target in phenotype‐related vascular disorders.

## Conflicts of interest

The authors declare no conflict of interest.

## Author contributions

JZ and JRG contributed equally to this study. YW, XG, and YF designed the experiments. JZ, JRG, and XLW performed the experiments. XW analyzed data. YF wrote the manuscript. XG and YF made manuscript revisions. YW supervised the study.

## Supporting information


**Fig S1.** Representative Western blot of SMMHC, SMA, calponin, tagln expression in SMCs treated with 25ng/ml PDGF (platelet‐derived growth factor)‐BB. Statistical analyses were performed using two‐tailed Student’s t test.
**Fig S2.** Phase and immunofluorescence confocal images revealed p68 (red) expression in control and injured rat carotid arteries.
**Fig S3.** Human SMCs transfected by GFP or TWIST1 Virus were treated with 10 nmol/mL MG132 and then subjected to Western blot analysis.

## Data Availability

The data are available from the corresponding author upon reasonable request.

## References

[1] Murgai M , Ju W , Eason M , Kline J , Beury DW , Kaczanowska S , Miettinen MM , Kruhlak M , Lei H , Shern JF *et al*, (2017) KLF4‐dependent perivascular cell plasticity mediates pre‐metastatic niche formation and metastasis. Nat Med 23, 1176–1190. 28920957 10.1038/nm.4400 PMC5724390

[2] Fan Y , Zhang J , Chen CY , Xiao YB , Asico LD , Jose PA , Xu JC , Qian GS and Zeng CY (2017) Macrophage migration inhibitory factor triggers vascular smooth muscle cell dedifferentiation by a p68‐serum response factor axis. Cardiovasc Res 113, 519–530. 28165114 10.1093/cvr/cvx025

[3] Shi JH , Zheng B , Li YH , Sun Y , Han AL , Zhang XH , Lv XR , Chen S and Wen JK (2013) Novel insight into Y‐box binding protein 1 in the regulation of vascular smooth muscle cell proliferation through targeting GC box‐dependent genes. FEBS Lett 587, 1326–1332. 23499936 10.1016/j.febslet.2013.02.047

[4] Zhao G , Fu Y , Cai Z , Yu F , Gong Z , Dai R , Hu Y , Zeng L , Xu Q and Kong W (2017) Unspliced XBP1 confers VSMC homeostasis and prevents aortic aneurysm formation via FoxO4 interaction. Circ Res 121, 1331–1345. 29089350 10.1161/CIRCRESAHA.117.311450

[5] Harper KL , Sosa MS , Entenberg D , Hosseini H , Cheung JF , Nobre R , Avivar‐Valderas A , Nagi C , Girnius N , Davis RJ *et al*, (2016) Mechanism of early dissemination and metastasis in Her2+ mammary cancer. Nature 540, 588–592. 27974798 10.1038/nature20609 PMC5471138

[6] van Doorn R , Dijkman R , Vermeer MH , Out‐Luiting JJ , van der Raaij‐Helmer EM , Willemze R and Tensen CP (2004) Aberrant expression of the tyrosine kinase receptor EphA4 and the transcription factor twist in Sézary syndrome identified by gene expression analysis. Cancer Res 64, 5578–5586. 15313894 10.1158/0008-5472.CAN-04-1253

[7] Nurnberg ST , Guerraty MA , Wirka RC , Rao HS , Pjanic M , Norton S , Serrano F , Perisic L , Elwyn S , Pluta J *et al*, (2020) Genomic profiling of human vascular cells identifies TWIST1 as a causal gene for common vascular diseases. PLoS Genet 16, e1008538. 10.1371/journal.pgen.1008538 PMC6975560 31917787

[8] Mahmoud MM , Kim HR , Xing R , Hsiao S , Mammoto A , Chen J , Serbanovic‐Canic J , Feng S , Bowden NP , Maguire R *et al*, (2016) TWIST1 integrates endothelial responses to flow in vascular dysfunction and atherosclerosis. Circ Res 2016, 450–462. 10.1161/CIRCRESAHA.116.308870 PMC4959828 27245171

[9] Fan Y , Gu X , Zhang J , Sinn K , Klepetko W , Wu N , Foris V , Solymosi P , Kwapiszewska G and Kuebler WM . (2020) TWIST1 Drives Smooth Muscle Cell Proliferation in Pulmonary Hypertension via Loss of GATA‐6 and BMPR2. Am J Respir Crit Care Med 202, 1283–1296. 32692930 10.1164/rccm.201909-1884OC

[10] Fan Y , Chen Y , Zhang J , Yang F , Hu Y , Zhang L , Zeng C and Xu Q (2019) Protective role of RNA helicase DEAD‐Box protein 5 in smooth muscle cell proliferation and vascular remodeling. Circ Res 124, e84–e100. 30879402 10.1161/CIRCRESAHA.119.314062

[11] Lu Y , Thavarajah T , Gu W , Cai J and Xu Q (2018) Impact of miRNA in atherosclerosis. Arterioscler Thromb Vasc Biol 38, e159–e170. 30354259 10.1161/ATVBAHA.118.310227 PMC6795547

[12] Maegdefessel L , Rayner KJ and Leeper NJ (2015) MicroRNA regulation of vascular smooth muscle function and phenotype: early career committee contribution. Arterioscler Thromb Vasc Biol 35, 2–6. 25520518 10.1161/ATVBAHA.114.304877

[13] Kang H and Hata A (2012) MicroRNA regulation of smooth muscle gene expression and phenotype. Curr Opin Hematol 19, 224–231. 22406821 10.1097/MOH.0b013e3283523e57 PMC5226630

[14] Rangrez AY , Massy ZA , Metzinger‐Le Meuth V and Metzinger L (2011) miR‐143 and miR‐145: molecular keys to switch the phenotype of vascular smooth muscle cells. Circ Cardiovasc Genet 4, 197–205. 21505201 10.1161/CIRCGENETICS.110.958702

[15] Zhao W , Zhao SP and Zhao YH (2015) MicroRNA‐143/‐145 in Cardiovascular diseases. Biomed Res Int 2015, 531740. 26221598 10.1155/2015/531740 PMC4499377

[16] Cheng Y , Liu X , Yang J , Lin Y , Xu DZ , Lu Q , Deitch EA , Huo Y , Delphin ES and Zhang C (2009) MicroRNA‐145, a novel smooth muscle cell phenotypic marker and modulator, controls vascular neointimal lesion formation. Circ Res 105, 158–166. 19542014 10.1161/CIRCRESAHA.109.197517 PMC2728297

[17] Baumgarten A , Bang C , Tschirner A , Engelmann A , Adams V , von Haehling S , Doehner W , Pregla R , Anker MS , Blecharz K *et al*, (2013) TWIST1 regulates the activity of ubiquitin proteasome system via the miR‐199/214 cluster in human end‐stage dilated cardiomyopathy. Int J Cardiol 168, 1447–1452. 23360823 10.1016/j.ijcard.2012.12.094

[18] Simpson P (1983) Maternal‐Zygotic gene interactions during formation of the dorsoventral pattern in Drosophila embryos. Genetics 105, 615–632. 17246169 10.1093/genetics/105.3.615 PMC1202177

[19] Füchtbauer EM (1995) Expression of M‐twist during postimplantation development of the mouse. Dev Dyn 204, 316–322. 8573722 10.1002/aja.1002040309

[20] Zheng X , Carstens JL , Kim J , Scheible M , Kaye J , Sugimoto H , Wu CC , LeBleu VS and Kalluri R (2015) Epithelial‐to‐mesenchymal transition is dispensable for metastasis but induces chemoresistance in pancreatic cancer. Nature 527, 525–530. 26560028 10.1038/nature16064 PMC4849281

[21] Ma L , Teruya‐Feldstein J and Weinberg RA (2007) Tumour invasion and metastasis initiated by microRNA‐10b in breast cancer. Nature 449, 682–688. 17898713 10.1038/nature06174

[22] Kumar MS , Hendrix JA , Johnson AD and Owens GK (2003) Smooth muscle alpha‐actin gene requires two E‐boxes for proper expression in vivo and is a target of class I basic helix‐loop‐helix proteins. Circ Res 92, 840–847. 12663487 10.1161/01.RES.0000069031.55281.7C

[23] Small EM and Olson EN (2011) Pervasive roles of microRNAs in cardiovascular biology. Nature 469, 336–342. 21248840 10.1038/nature09783 PMC3073349

[24] Small EM , Frost RJ and Olson EN (2010) MicroRNAs add a new dimension to cardiovascular disease. Circulation 121, 1022–1032. 20194875 10.1161/CIRCULATIONAHA.109.889048 PMC2847432

[25] Lekka E and Hall J (2018) Noncoding RNAs in disease. FEBS Lett 592, 2884–2900. 29972883 10.1002/1873-3468.13182 PMC6174949

[26] Zeng Y , Pan Y , Liu H , Kang K , Wu Y , Hui G , Peng W , Ramchandran R , Raj JU and Gou D (2014) MiR‐20a regulates the PRKG1 gene by targeting its coding region in pulmonary arterial smooth muscle cells. FEBS Lett 588, 4677–4685. 25447536 10.1016/j.febslet.2014.10.040

[27] Kee HJ , Park S , Kwon JS , Choe N , Ahn Y , Kook H and Jeong MH (2013) B cell translocation gene, a direct target of miR‐142‐5p, inhibits vascular smooth muscle cell proliferation by down‐regulating cell cycle progression. FEBS Lett 587, 2385–2392. 23770100 10.1016/j.febslet.2013.06.005

[28] Cordes KR , Sheehy NT , White MP , Berry EC , Morton SU , Muth AN , Lee TH , Miano JM , Ivey KN and Srivastava D (2009) miR‐145 and miR‐143 regulate smooth muscle cell fate and plasticity. Nature 460, 705–710. 19578358 10.1038/nature08195 PMC2769203

[29] Li P , Zhu N , Yi B , Wang N , Chen M , You X , Zhao X , Solomides CC , Qin Y and Sun J (2013) MicroRNA‐663 regulates human vascular smooth muscle cell phenotypic switch and vascular neointimal formation. Circ Res 113, 1117–1127. 24014830 10.1161/CIRCRESAHA.113.301306 PMC4537615

[30] Torella D , Iaconetti C , Catalucci D , Ellison GM , Leone A , Waring CD , Bochicchio A , Vicinanza C , Aquila I , Curcio A *et al*, (2011) MicroRNA‐133 controls vascular smooth muscle cell phenotypic switch in vitro and vascular remodeling in vivo. Circ Res 109, 880–893. 21852550 10.1161/CIRCRESAHA.111.240150

[31] Davis BN , Hilyard AC , Lagna G and Hata A (2008) SMAD proteins control DROSHA‐mediated microRNA maturation. Nature 454, 56–61. 18548003 10.1038/nature07086 PMC2653422

[32] Fuller‐Pace FV and Moore HC (2011) RNA helicases p68 and p72: multifunctional proteins with important implications for cancer development. Future Oncol 7, 239–251. 21345143 10.2217/fon.11.1

