# Supplementary material for: TWIST1 induces phenotypic switching of vascular smooth muscle cells by downregulating p68 and microRNA‐143/145
Source: FEBS Open Bio. 2021 Feb 3;11(3):932–43. doi: 10.1002/2211-5463.13092 (PMC7931233; doi:10.1002/2211-5463.13092)
Supplement: Supplementary file 1 — Fig S1. Representative Western blot of SMMHC, SMA, calponin, tagln expression in SMCs treated with 25ng/ml PDGF (platelet‐derived growth factor)‐BB. Statistical analyses were performed using two‐tailed Student’s t test. Fig S2. Phase and immunofluorescence confocal images revealed p68 (red) expression in control and injured rat carotid arteries. Fig S3. Human SMCs transfected by GFP or TWIST1 Virus were treated with 10 nmol/mL MG132 and then subjected to Western blot analysis. [file FEB4-11-932-s001.pdf]

Supplemental Figure 1

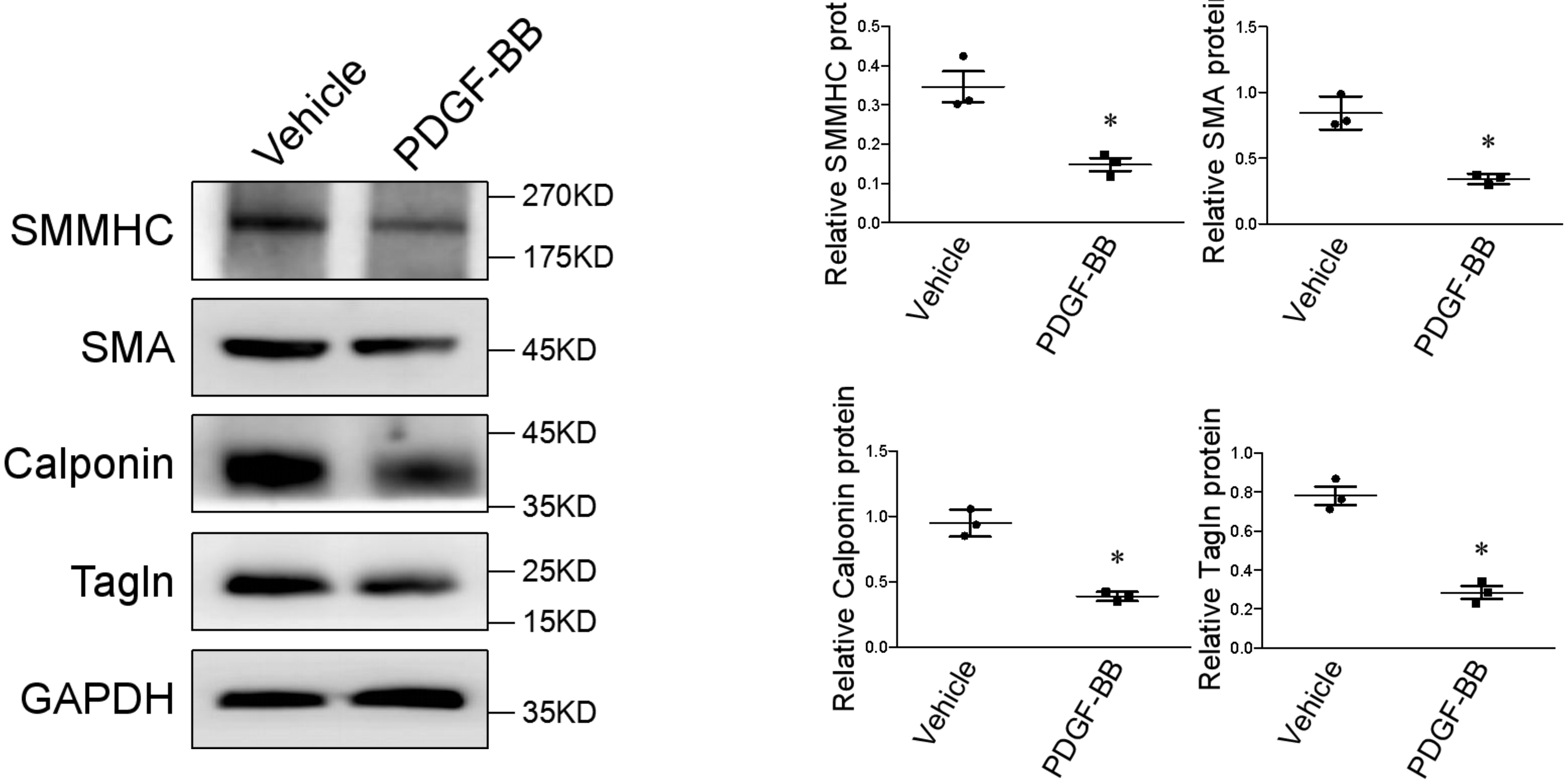

Supplemental figure 1. Representative Western blot of SMMHC, SMA, calponin, tagln expression in SMCs treated with 25ng/ml PDGF (platelet-derived growth factor)-BB. *P<0.05.

Supplemental Figure 2

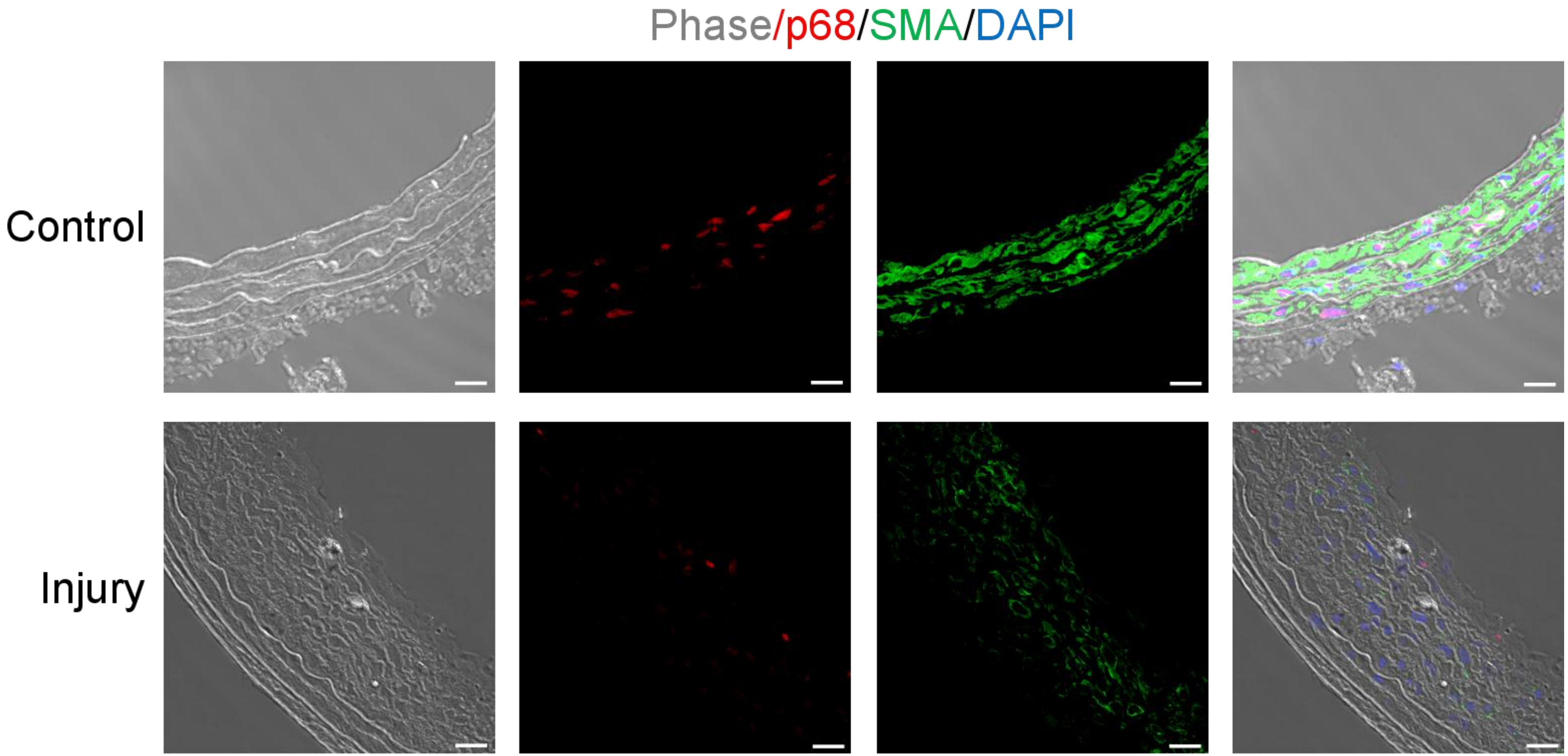

Supplemental figure 2. Phase and immunofluorescence confocal images revealed p68 (red) expression in control and injured rat carotid arteries. Anti-SMA staining for SMA (green). Cell nuclei were stained with DAPI (blue). Scale bar = 20 μm.

Supplemental Figure 3

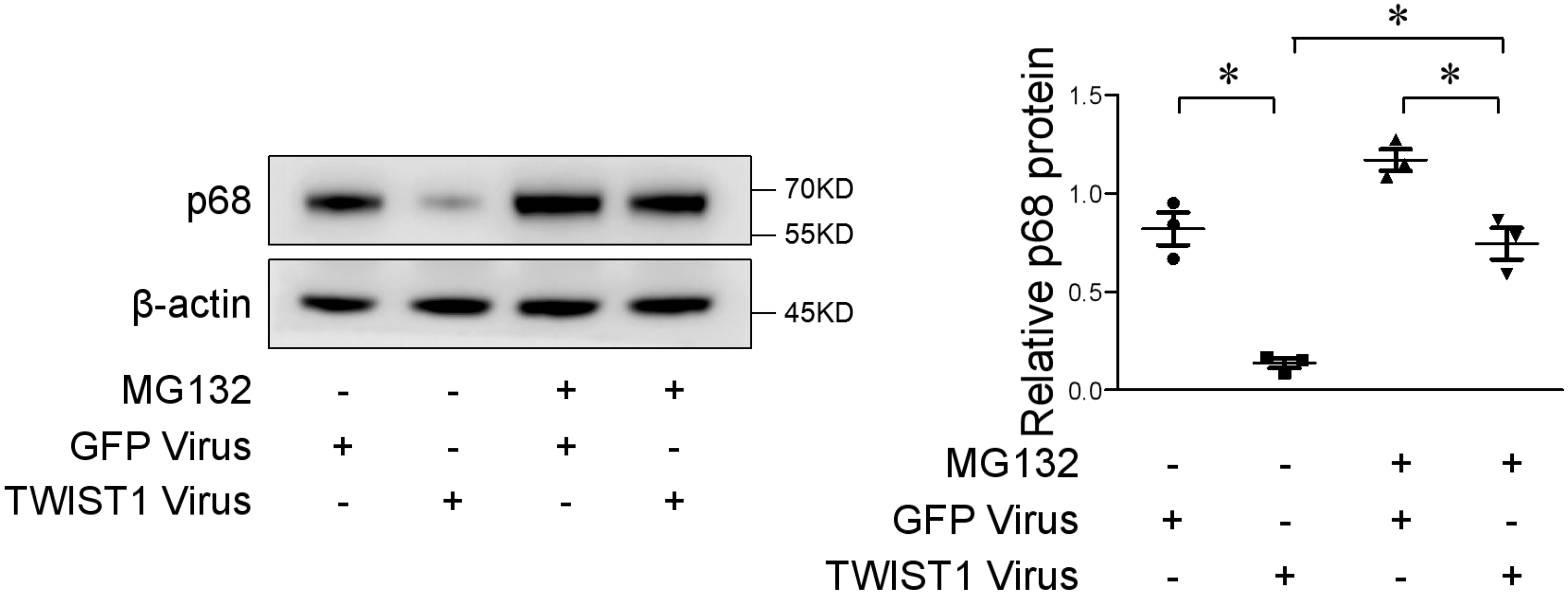

Supplemental figure 3. Human SMCs transfected by GFP or TWIST1 Virus were treated with 10 nmol/mL MG132 and then subjected to Western blot analysis. *P<0.05.
